# Trends in Socioeconomic Inequalities in the Use of Formal Health Services Among Peruvian Adults in Need of Care: 2015–2024

**DOI:** 10.5334/aogh.5481

**Published:** 2026-11-10

**Authors:** Miguel A. Arce-Huamani, Kosseth Otero-Krugger, Gustavo A. Caceres-Cuellar, Anyela Y. Guevara-Paz, Cleofe R. Lopez-Quispe, Uriel Palacios-Barahona, Gabriel De la Cruz-Ku, J. Smith Torres-Roman, Luis M. Tasayco-Marquez

**Affiliations:** 1 Public Health, Surveillance and Applied Research, Programa Academico de Medicina Humana, Facultad de Ciencias de la Salud, Universidad Privada Norbert Wiener, Lima 15046, Peru; 2 Cancer and Hematology Institute, Hospital Universitario Mayor Méderi, Universidad del Rosario, Bogotá, Colombia; 3 Universidad Cientifica del Sur, Lima, Peru; 4 Escuela de Posgrado, Universidad Continental, Lima, Peru; 5 Escuela de Posgrado, Universidad Tecnologica del Peru, Lima, Peru

**Keywords:** healthcare utilisation, socioeconomic inequalities, poverty, health services accessibility, universal health coverage

## Abstract

*Background:* Although health insurance coverage has expanded in Peru, equitable access to formal healthcare remains uncertain. We examined 2015–2024 trends and poverty-related inequalities in formal service use among adults reporting recent health problems.

*Methods:* We analysed 10 annual rounds of Peru’s National Household Survey in a repeated cross-sectional study. Eligible adults were aged ≥18 years and reported a health problem in the preceding four weeks. We excluded those who considered care unnecessary and those using both a healthcare facility and a pharmacy. Survey-weighted prevalence and annual trends were estimated. Survey-adjusted modified Poisson models yielded adjusted prevalence ratios (aPRs). A poverty-by-period interaction compared 2015–2019, 2020–2021, and 2022–2024.

*Results:* Among 330,165 adults, 41.2% used formal health services. Utilisation declined 2.00% annually (95% CI, −2.25 to −1.75), dropped sharply in 2020, and recovered incompletely. Compared with non-poor adults, use was lower in non-extreme poverty (aPR, 0.80; 95% CI, 0.79–0.82) and extreme poverty (aPR, 0.74; 95% CI, 0.71–0.77). The poverty-by-period interaction was significant (P < 0.001), with the widest inequalities in 2020–2021 (aPRs, 0.74 and 0.61). Indigenous-language speakers and Highlands residents had lower utilisation. Insurance was associated with greater use, but inequalities persisted.

*Conclusion:* Formal healthcare use declined while socioeconomic inequalities widened during the pandemic. Equitable coverage requires addressing financial, territorial, and cultural barriers beyond insurance enrolment.

## Background

Universal health coverage requires more than insurance enrolment. It also depends on whether people can obtain healthcare when they need it. Although the global effective-coverage index increased from 45.8 in 1990 to 60.3 in 2019, substantial differences persisted across countries [1]. In Latin America, studies from Ecuador and other countries have documented socioeconomic inequalities in healthcare utilisation and barriers to seeking care [2, 3]. Even where services are guaranteed, some eligible individuals do not use them, as reported in Chile [4]. During the COVID-19 pandemic, healthcare disruptions were documented across 14 Latin American and Caribbean countries [5]. Evidence from Peru has also linked ethnicity-related mistreatment to the non-use of health services [6]. Differences in healthcare coverage and quality across health-system subsystems further suggest that formal entitlement does not necessarily ensure equitable access [7]. These findings highlight the importance of examining actual service use, particularly among people reporting a need for care.

Peru provides an important setting for examining this gap. Despite improvements in several health indicators, its healthcare system continues to face fragmentation, underinvestment, and unequal access to primary and culturally appropriate care [8, 9]. In 2019, only 25.4% (95% CI, 24.8–26.1) of adults reporting a recent health problem received a physician consultation, with higher utilisation among wealthier groups [10]. Access to the Comprehensive Health Insurance scheme (Seguro Integral de Salud, SIS) has been associated with increased service use, particularly among older adults and people with lower educational attainment, although the estimated gains were not statistically significant in rural areas [11]. Geographic and ethnic inequalities present additional challenges. Among 1,043 Indigenous communities in the Peruvian Amazon, 15.44% were located more than eight hours from the nearest health facility [12], while disparities in cancer screening have also been documented across regions and population groups [13]. Together, these findings indicate that insurance coverage may improve healthcare utilisation without fully addressing the socioeconomic and territorial barriers that influence access.

The COVID-19 pandemic placed additional pressure on these existing inequalities. In 2020, 52.2% of Peruvians reporting symptoms consistent with COVID-19 did not use health services, with particularly high non-use in areas of the Amazon region [14]. Evidence from 25 developing countries suggested some recovery in healthcare seeking between 2020 and 2021 [15]. However, studies conducted in Peru documented substantial reductions in maternal and child health services [16] and a marked increase in unmet healthcare needs during lockdown [17]. Additional evidence among female paid domestic workers showed that pandemic-related changes in healthcare seeking were not evenly distributed across social groups [18]. These findings suggest that the pandemic may have reinforced existing inequalities in access to formal healthcare, making it important to examine whether utilisation recovered similarly across socioeconomic groups.

Previous ENAHO research has described national trends and factors associated with the non-use of formal health services between 2015 and 2024 [19]. However, the extent to which inequalities in formal healthcare utilisation between non-poor, non-extreme poor, and extreme poor adults changed across the pre-pandemic, pandemic, and recovery periods warrants closer examination. Assessing these differences among adults reporting a recent health problem is particularly relevant because insurance coverage and actual healthcare utilisation represent distinct dimensions of access. This study aimed to examine trends in formal health service use and quantify socioeconomic inequalities among Peruvian adults reporting a need for care between 2015 and 2024.

## Materials and Methods

### Study design and setting

We conducted a repeated cross-sectional analysis using secondary microdata from the Peruvian National Household Survey on Living Conditions and Poverty (Encuesta Nacional de Hogares, ENAHO), collected annually from 2015 to 2024 by the National Institute of Statistics and Informatics of Peru (Instituto Nacional de Estadística e Informática, INEI). Each ENAHO round constitutes an independent, nationally representative cross-section; therefore, all trend estimates reflect population-level changes across calendar years rather than within-person longitudinal changes.

ENAHO uses a probabilistic, stratified, multistage cluster sampling design and provides national, departmental, urban/rural, and geographic-domain representativeness. Primary sampling units are clusters of private dwellings in urban areas and rural census areas in rural strata, while households constitute the second-stage sampling units. Standardised face-to-face interviews are conducted by trained fieldworkers using structured questionnaires and electronic data capture.

The unit of analysis was the adult person-year observation recorded in the ENAHO health module, with eligibility determined by the age, recent morbidity, and care-seeking criteria described below. Annual ENAHO files from 2015 through 2024 were pooled into a single harmonised dataset. All descriptive estimates, trend analyses, bivariate analyses, and regression models incorporated the complex survey design using the individual expansion factor, sampling strata, and primary sampling units.

### Population and sample

The source population comprised adults aged 18 years or older living in private households in Peru. The pooled 2015–2024 ENAHO health-module files contained 1,201,908 person-level records. After excluding 387,318 records from individuals younger than 18 years, 814,590 adult observations remained. Among these adults, 331,437 had no affirmative response to the items assessing symptoms, illness, relapse of a chronic condition, or an accident during the preceding four weeks (p4021–p4024), leaving 483,153 observations with a recent health problem. We then excluded 149,866 observations from respondents who reported not attending a health facility because their condition was not serious or care was unnecessary (p4095). To compare exclusive care-seeking patterns, we also excluded 3,122 observations from respondents who reported using both a formal healthcare facility and a pharmacy during the four-week reference period, as the survey did not establish the sequence of use. The final analytic sample comprised 330,165 adult observations across the 10 survey rounds. All counts are unweighted and refer to person-year observations from independent annual survey rounds rather than unique individuals followed over time.

### Data collection

ENAHO data are collected annually by INEI through standardised household interviews. Trained interviewers administer structured questionnaires covering sociodemographic characteristics, education, employment, poverty, household conditions, health insurance, health problems, chronic conditions, disability, and healthcare utilisation. The health module includes information on recent morbidity, care-seeking behaviour, place of care, type of provider, reasons for not seeking care, and insurance affiliation.

For the present analysis, we used harmonised variables available across the 2015–2024 ENAHO rounds. These included survey year, recent health problem or recent need for care, IPRESS utilisation, household poverty status, sex, age, educational level, marital status, mother tongue/language, type of health insurance, chronic illness or discomfort, disability, area of residence, natural region, department, sampling weight, stratum, and cluster.

Fieldwork procedures are implemented by INEI using standardised protocols, interviewer training, supervision, consistency checks, and data quality controls to ensure comparability across survey years. The present study analysed only de-identified public-use microdata.

### Variables

The dependent variable was the use of formal health services, operationalised as IPRESS utilisation among adults reporting a recent health problem or recent need for care. In Peru, IPRESS refers to Health Service Provider Institutions (Instituciones Prestadoras de Servicios de Salud), including public, social security, Armed Forces/Police, and private healthcare facilities. In the harmonised analytic dataset, the variable nusfps was coded as 0 for respondents who used an IPRESS and 1 for those who did not. Throughout the manuscript, the outcome of interest is IPRESS utilisation, with non-use as the comparison category.

The main independent variable was household poverty status, based on the official INEI household poverty classification. It was categorised into three groups: non-poor, non-extreme poor, and extreme poor. The non-poor group was used as the reference category in all regression models.

The time variable was survey year, ranging from 2015 to 2024. For period-based analyses, survey year was grouped into three analytical periods: 2015–2019, representing the pre-pandemic period; 2020–2021, representing the pandemic-disruption period; and 2022–2024, representing the post-2022 partial recovery period. These periods were used to assess whether socioeconomic inequalities in IPRESS use changed across key stages of health-system disruption and recovery.

Covariates were selected a priori based on social determinants of health and health services utilisation frameworks. Sociodemographic covariates included sex, age group, educational level, marital status, and mother tongue/language. Age was categorised as 18–29, 30–39, 40–59, and ≥60 years. Educational level was categorised as no formal education, primary, secondary, and higher education. Marital status was categorised as single, cohabiting, married, widowed, and divorced/separated. Mother tongue/language was categorised as Spanish and Quechua, Aymara, or other indigenous language.

Enabling and health-system variables included type of health insurance, categorised as uninsured, EsSalud, Seguro Integral de Salud (SIS), Armed Forces/Police insurance, and private or other insurance. Because health insurance may partly mediate the association between poverty and use of formal health services, two multivariable models were specified: one excluding type of health insurance and another additionally adjusting for type of health insurance.

Need-related covariates included self-reported chronic illness or discomfort and disability. Territorial covariates included area of residence, categorised as urban or rural, and natural region, categorised as Coast, Highlands, Rainforest, and Metropolitan Lima. Department was derived from UBIGEO and used in a sensitivity analysis as an alternative territorial adjustment.

The operational definitions, original ENAHO variables, harmonisation rules, coding, and reference categories are presented in Supplementary Table S1.

### Data analysis

All analyses accounted for the complex ENAHO survey design. Sampling weights were applied using the individual expansion factor facpob07. Sampling strata and primary sampling units were incorporated to account for stratification and clustering. Because annual survey files were pooled, survey design identifiers were handled consistently across years to preserve the repeated cross-sectional structure of the dataset.

First, we described the study population using unweighted frequencies and survey-weighted percentages. These results are presented in Table 1. All percentages were calculated using the ENAHO sampling weight, and the denominator was restricted to adults aged 18 years or older reporting a recent health problem or recent need for care.

**Table 1:** Characteristics of Peruvian adults reporting a recent health problem, ENAHO 2015–2024.

GENERAL CHARACTERISTICS	N (= 330,165)	WEIGHTED %
**SOCIODEMOGRAPHIC CHARACTERISTICS**		
**Sex**		
Male	135,547	40.5
Female	194,618	59.5
**Age group**		
18–29 years	55,964	17.8
30–39 years	52,157	15.9
40–59 years	119,932	36.3
≥60 years	102,112	29.9
**Educational level**		
No formal education	31,828	8.4
Primary	147,578	41.6
Secondary	84,467	29.1
Higher	66,002	20.8
**Marital status**		
Single	56,021	18.8
Cohabiting	88,254	26.1
Married	114,409	33.2
Widowed	31,391	9.2
Divorced/Separated	40,090	12.7
**Mother tongue/language**		
Spanish	246,996	75.9
Quechua/Aymara/Other indigenous	83,115	24.1
**SOCIOECONOMIC CHARACTERISTICS**		
**Household poverty status**		
Non-poor	262,771	79.7
Non-extreme poor	54,486	16.9
Extreme poor	12,908	3.4
**HEALTH INSURANCE**		
**Type of health insurance**		
Uninsured	57,526	18.5
EsSalud	78,118	25.6
SIS	188,211	53.5
Armed Forces/Police	3,436	1.3
Private/Other	2,874	1.1
**CLINICAL/HEALTH-RELATED CHARACTERISTICS**		
**Chronic illness or discomfort**		
No	114,644	33.9
Yes	215,521	66.1
**Disability**		
No	298,454	90.6
Yes	31,711	9.4
**TERRITORIAL CHARACTERISTICS**		
**Area of residence**		
Urban	220,096	78.9
Rural	110,069	21.1
**Natural region**		
Coast	94,457	22.3
Highlands	139,212	38.1
Rainforest	62,569	11.2
Metropolitan Lima	33,927	28.5
**MAIN OUTCOME**		
**IPRESS utilisation**		
Did not use formal health services/IPRESS	193,225	58.8
Used formal health services/IPRESS	136,940	41.2

*Note*: The denominator is restricted to adults aged ≥18 years who reported a recent health problem or recent need for care. N values are unweighted counts; percentages are weighted using the ENAHO individual expansion factor facpob07. Estimates account for the complex ENAHO survey design using sampling weights, strata, and clusters. Variables with missing data: educational level, n = 290; mother tongue/language, n = 54. IPRESS: Health Service Provider Institution. EsSalud: Social Health Insurance. SIS: Comprehensive Health Insurance.

*Source*: National Household Survey on Living Conditions and Poverty, ENAHO 2015–2024.

We also conducted an additional descriptive analysis of care-seeking pathways among adults who did not use an IPRESS. Responses to the original ENAHO place-of-care items were classified into five mutually exclusive categories: no care sought, pharmacy or drugstore only, care at home only, another place of care only, and multiple non-IPRESS care options. We report unweighted frequencies and survey-weighted percentages using the same analytic sample and annual sampling weights.

Second, we estimated the weighted annual prevalence of IPRESS use from 2015 to 2024 overall and according to household poverty status. These estimates were plotted in Figure 1. Temporal change was summarised using log-linear models of annual weighted prevalence estimates. The annual relative change was reported with 95% confidence intervals. Because Joinpoint regression was not used for the main trend estimate, the term average annual percent change was not used.

**Figure 1:**
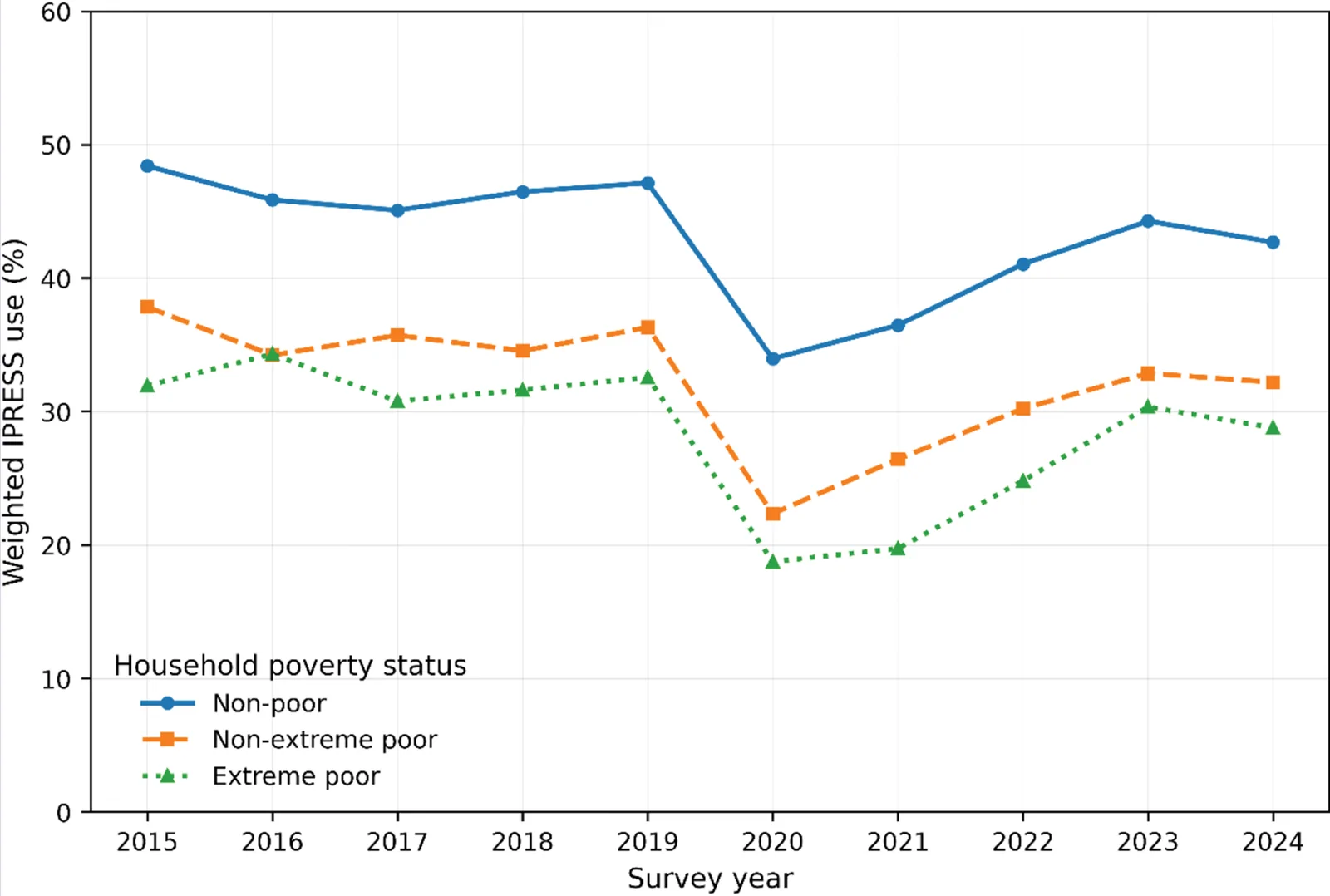
National trend in IPRESS use by poverty status among Peruvian adults reporting a recent health problem, ENAHO 2015–2024. Weighted annual prevalence estimates were calculated using the ENAHO individual expansion factor. The denominator was restricted to adults aged ≥18 years with recent need for care. The annual relative change was estimated from a log-linear model. Overall IPRESS use decreased by −2.00% per year (95% CI: −2.25 to −1.75). The estimated annual relative change was −1.69% among non-poor adults (95% CI: −1.95 to −1.43), −2.10% among non-extreme poor adults (95% CI: −2.75 to −1.43), and −2.13% among adults in extreme poverty (95% CI: −3.40 to −0.85).

Third, bivariate analyses were conducted to estimate the weighted prevalence of IPRESS use within each category of the explanatory variables. Crude prevalence ratios and 95% confidence intervals were estimated using survey-adjusted modified Poisson regression. These results are presented in Table 2 as weighted IPRESS use percentage, crude prevalence ratio, 95% confidence interval, and p-value. This approach was selected because it directly describes the prevalence of IPRESS use within each subgroup, rather than only the distribution of characteristics among users and non-users.

**Table 2:** Bivariate prevalence of IPRESS use among Peruvian adults reporting a recent health problem, ENAHO 2015–2024.

VARIABLE	WEIGHTED IPRESS USE (%)	CRUDE PR	95% CI	P-VALUE
**Household poverty status**				
Non-poor	43.6	Reference	Reference	
Non-extreme poor	32.3	0.74	0.72–0.75	<0.001
Extreme poor	28.5	0.65	0.63–0.68	<0.001
**Sex**				
Male	38.1	Reference	Reference	
Female	43.3	1.14	1.12–1.15	<0.001
**Age group**				
18–29 years	36.9	Reference	Reference	
30–39 years	38.8	1.05	1.03–1.07	<0.001
40–59 years	40.1	1.08	1.07–1.10	<0.001
≥60 years	46.3	1.25	1.23–1.28	<0.001
**Educational level**				
No formal education	38.6	Reference	Reference	
Primary	39.7	1.03	1.01–1.05	0.010
Secondary	40.1	1.04	1.02–1.06	<0.001
Higher	46.7	1.21	1.18–1.24	<0.001
**Marital status**				
Single	37.8	Reference	Reference	
Cohabiting	38.3	1.01	0.99–1.03	0.172
Married	43.5	1.15	1.13–1.17	<0.001
Widowed	45.7	1.21	1.18–1.24	<0.001
Divorced/Separated	42.6	1.13	1.10–1.15	<0.001
**Mother tongue/language**				
Spanish	42.4	Reference	Reference	
Quechua/Aymara/Other indigenous	37.3	0.88	0.87–0.89	<0.001
**Type of health insurance**				
Uninsured	23.9	Reference	Reference	
EsSalud	53.3	2.23	2.18–2.29	<0.001
SIS	40.7	1.71	1.66–1.75	<0.001
Armed Forces/Police	56.1	2.35	2.24–2.47	<0.001
Private/Other	56.8	2.38	2.26–2.51	<0.001
**Chronic illness or discomfort**				
No	35.0	Reference	Reference	
Yes	44.4	1.27	1.25–1.28	<0.001
**Disability**				
No	41.0	Reference	Reference	
Yes	42.6	1.04	1.02–1.06	<0.001
**Area of residence**				
Urban	42.4	Reference	Reference	
Rural	36.6	0.86	0.85–0.88	<0.001
**Natural region**				
Coast	41.8	Reference	Reference	
Highlands	35.3	0.84	0.83–0.86	<0.001
Rainforest	40.7	0.97	0.95–0.99	0.006
Metropolitan Lima	48.7	1.17	1.14–1.19	<0.001

*Note*: The denominator is restricted to adults aged ≥18 years reporting a recent health problem or recent need for care. Weighted IPRESS use percentages were calculated with facpob07. Crude PRs were estimated using modified Poisson regression. Bivariate tests and confidence intervals accounted for the ENAHO complex survey design, including sampling weights, strata, and clustered primary sampling units. PR: prevalence ratio; CI: confidence interval.

*Source*: ENAHO 2015–2024.

Fourth, multivariable associations between household poverty status and IPRESS use were estimated using modified Poisson regression models with log link and robust variance, incorporating the complex survey design. This modelling strategy was used because IPRESS use was a frequent binary outcome, and odds ratios would overestimate the magnitude of association. Results are reported as prevalence ratios with 95% confidence intervals and p-values.

Two adjusted models were specified. Model 1 adjusted for sex, age group, educational level, marital status, mother tongue/language, chronic illness or discomfort, disability, area of residence, natural region, and survey year, but did not include health insurance. Model 2 included the same covariates as Model 1 and additionally adjusted for type of health insurance. This two-model approach was used because health insurance may be a partial mediator between socioeconomic position and use of formal health services. Therefore, comparing Model 1 and Model 2 allowed us to evaluate whether the poverty gradient was attenuated after accounting for insurance affiliation.

Fifth, to formally evaluate changes in socioeconomic inequalities over time, we fitted a modified Poisson regression model including an interaction term between household poverty status and survey period. The model adjusted for sex, age group, educational level, marital status, mother tongue/language, type of health insurance, chronic illness or discomfort, disability, area of residence, and natural region. Adjusted prevalence ratios comparing non-extreme poor and extreme poor adults with non-poor adults were estimated within each survey period. The global p-value for the household poverty status × survey period interaction was used to assess whether socioeconomic inequalities differed across periods. These results are presented in Table 4.

**Table 3:** Multivariable analysis of factors associated with IPRESS use among Peruvian adults reporting a recent health problem, ENAHO 2015–2024.

ASSOCIATED FACTORS	CRUDE PR (95% CI)	P-VALUE	MODEL 1 aPR (95% CI)	P-VALUE	MODEL 2 aPR (95% CI)	P-VALUE
**Household poverty status**						
Non-poor	Reference		Reference		Reference	
Non-extreme poor	0.74 (0.72–0.75)	<0.001	0.80 (0.79–0.82)	<0.001	0.80 (0.79–0.82)	<0.001
Extreme poor	0.65 (0.63–0.68)	<0.001	0.75 (0.72–0.78)	<0.001	0.74 (0.71–0.77)	<0.001
**Sex**						
Male	Reference		Reference		Reference	
Female	1.14 (1.12–1.15)	<0.001	1.14 (1.12–1.15)	<0.001	1.11 (1.10–1.13)	<0.001
**Age group**						
18–29 years	Reference		Reference		Reference	
30–39 years	1.05 (1.03–1.07)	<0.001	1.03 (1.00–1.05)	0.018	1.01 (0.98–1.03)	0.520
40–59 years	1.08 (1.07–1.10)	<0.001	1.03 (1.00–1.05)	0.023	1.01 (0.98–1.03)	0.571
≥60 years	1.25 (1.23–1.28)	<0.001	1.19 (1.16–1.22)	<0.001	1.13 (1.10–1.16)	<0.001
**Educational level**						
No formal education	Reference		Reference		Reference	
Primary	1.03 (1.01–1.05)	0.010	1.07 (1.05–1.10)	<0.001	1.06 (1.04–1.09)	<0.001
Secondary	1.04 (1.02–1.06)	<0.001	1.12 (1.09–1.15)	<0.001	1.09 (1.06–1.11)	<0.001
Higher	1.21 (1.18–1.24)	<0.001	1.26 (1.23–1.30)	<0.001	1.16 (1.13–1.19)	<0.001
**Marital status**						
Single	Reference		Reference		Reference	
Cohabiting	1.01 (0.99–1.03)	0.172	1.08 (1.05–1.10)	<0.001	1.03 (1.01–1.05)	0.005
Married	1.15 (1.13–1.17)	<0.001	1.13 (1.11–1.16)	<0.001	1.05 (1.02–1.07)	<0.001
Widowed	1.21 (1.18–1.24)	<0.001	1.11 (1.08–1.14)	<0.001	1.04 (1.01–1.07)	0.003
Divorced/Separated	1.13 (1.10–1.15)	<0.001	1.07 (1.04–1.10)	<0.001	1.04 (1.02–1.07)	<0.001
**Mother tongue/language**						
Spanish	Reference		Reference		Reference	
Quechua/Aymara/Other indigenous	0.88 (0.87–0.89)	<0.001	0.97 (0.95–0.99)	<0.001	0.97 (0.95–0.99)	<0.001
**Type of health insurance**						
Uninsured	Reference		—		Reference	
EsSalud	2.23 (2.18–2.29)	<0.001	Not included	—	2.01 (1.96–2.06)	<0.001
SIS	1.71 (1.66–1.75)	<0.001	Not included	—	1.81 (1.77–1.85)	<0.001
Armed Forces/Police	2.35 (2.24–2.47)	<0.001	Not included	—	2.03 (1.94–2.14)	<0.001
Private/Other	2.38 (2.26–2.51)	<0.001	Not included	—	2.08 (1.97–2.19)	<0.001
**Chronic illness or discomfort**						
No	Reference		Reference		Reference	
Yes	1.27 (1.25–1.28)	<0.001	1.18 (1.16–1.20)	<0.001	1.16 (1.14–1.18)	<0.001
**Disability**						
No	Reference		Reference		Reference	
Yes	1.039 (1.019–1.060)	<0.001	0.995 (0.976–1.016)	0.659	0.981 (0.962–1.001)	0.057
**Area of residence**						
Urban	Reference		Reference		Reference	
Rural	0.86 (0.85–0.88)	<0.001	1.07 (1.05–1.08)	<0.001	1.06 (1.04–1.07)	<0.001
**Natural region**						
Coast	Reference		Reference		Reference	
Highlands	0.84 (0.83–0.86)	<0.001	0.88 (0.87–0.90)	<0.001	0.89 (0.87–0.90)	<0.001
Rainforest	0.97 (0.95–0.99)	0.006	1.02 (1.00–1.04)	0.031	1.01 (0.99–1.03)	0.242
Metropolitan Lima	1.17 (1.14–1.19)	<0.001	1.16 (1.14–1.18)	<0.001	1.14 (1.12–1.16)	<0.001

*Note*: The denominator is restricted to adults aged ≥18 years reporting a recent health problem or recent need for care. N values are unweighted. Crude and adjusted prevalence ratios were estimated using modified Poisson regression with robust variance and the ENAHO complex survey design. Model 1 adjusted for sex, age group, educational level, marital status, mother tongue/language, chronic illness or discomfort, disability, area of residence, natural region, and survey year. Model 2 additionally adjusted for type of health insurance. Disability estimates are reported with three decimals because the confidence interval borders the null value. PR: prevalence ratio; aPR: adjusted prevalence ratio; CI: confidence interval.

*Source*: ENAHO 2015–2024.

**Table 4:** Adjusted association between household poverty status and IPRESS use by survey period among Peruvian adults reporting a recent health problem, ENAHO 2015–2024.

SURVEY PERIOD	HOUSEHOLD POVERTY STATUS	aPR (95% CI)	P-VALUE
**2015–2019**	Non-poor	Reference	
	Non-extreme poor	0.83 (0.81–0.86)	<0.001
	Extreme poor	0.80 (0.76–0.84)	<0.001
**2020–2021**	Non-poor	Reference	
	Non-extreme poor	0.74 (0.70–0.78)	<0.001
	Extreme poor	0.61 (0.54–0.70)	<0.001
**2022–2024**	Non-poor	Reference	
	Non-extreme poor	0.78 (0.75–0.81)	<0.001
	Extreme poor	0.73 (0.68–0.79)	<0.001

*Note*: The denominator is restricted to adults aged ≥18 years reporting a recent health problem or recent need for care. Estimates were obtained from a modified Poisson regression model including the interaction between household poverty status and survey period. The model adjusted for sex, age group, educational level, marital status, mother tongue/language, type of health insurance, chronic illness or discomfort, disability, area of residence, and natural region. The global P-value for the poverty × period interaction was <0.001. aPR: adjusted prevalence ratio; CI: confidence interval.

*Source*: ENAHO 2015–2024.

Sixth, we estimated adjusted predicted prevalence of IPRESS use by household poverty status and survey period from the interaction model. These adjusted predictions and their 95% confidence intervals are shown in Figure 2. This analysis was used to present absolute adjusted differences in IPRESS utilisation across socioeconomic groups and periods.

**Figure 2:**
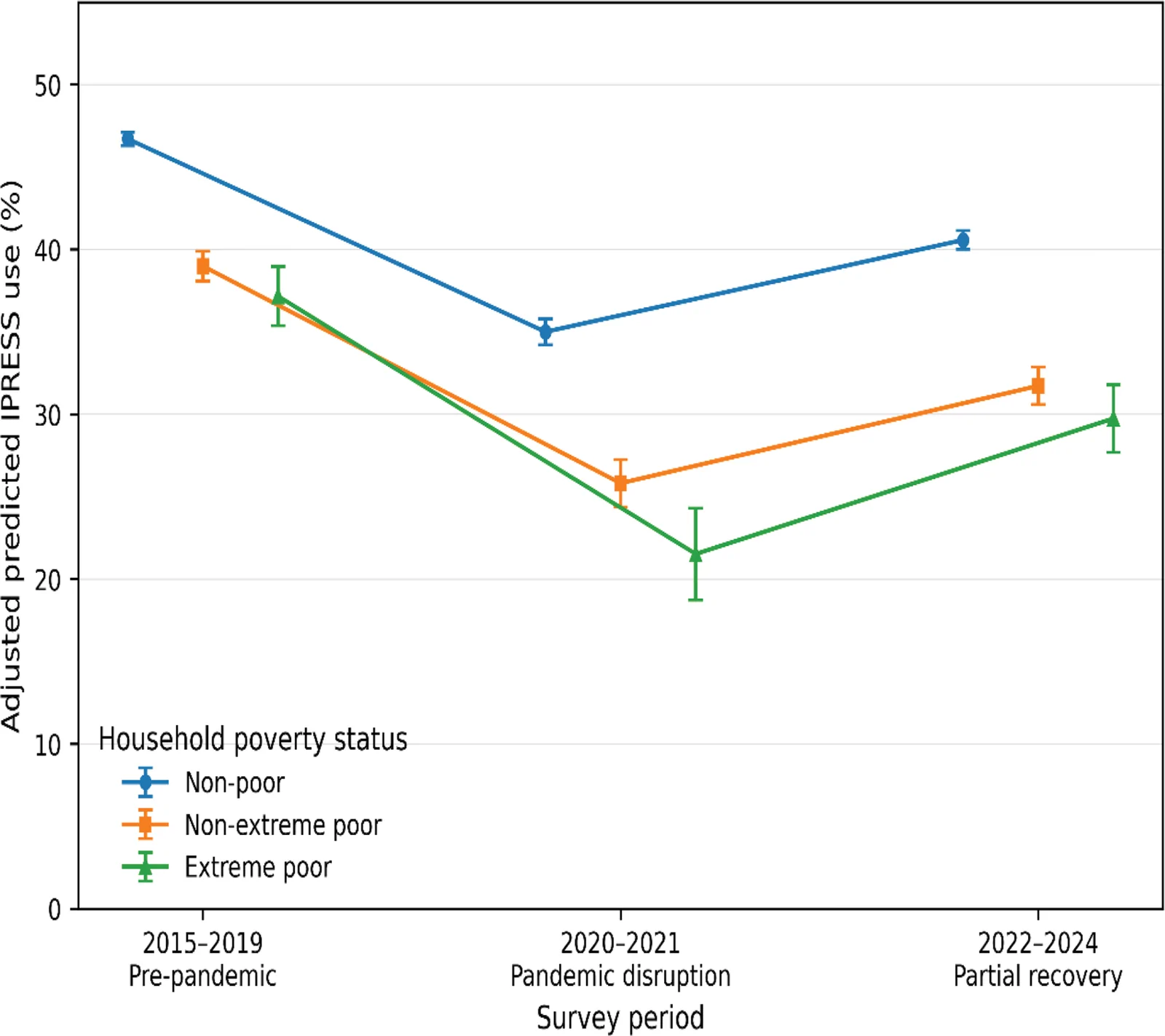
Adjusted predicted prevalence of IPRESS use by poverty status and survey period among Peruvian adults reporting a recent health problem, ENAHO 2015–2024. Predicted prevalences were estimated from a modified Poisson regression model including the interaction between household poverty status and survey period and adjusted for sex, age group, educational level, marital status, mother tongue/language, type of health insurance, chronic illness or discomfort, disability, area of residence, and natural region. Error bars represent 95% confidence intervals. The denominator was restricted to adults aged ≥18 years with recent need for care.

Seventh, supplementary analyses were performed to strengthen the temporal and equity interpretation. Supplementary Table S3 presents the adjusted association between survey period and IPRESS use. Supplementary Table S4 presents year-specific weighted prevalence of IPRESS use overall and by household poverty status. Supplementary Table S5 summarises weighted prevalence by period and poverty status. Supplementary Table S6 presents annual absolute and relative socioeconomic gaps in IPRESS use, including the absolute difference in percentage points and the relative ratio comparing non-poor adults with adults in extreme poverty. Supplementary Table S7 presents a sensitivity analysis replacing natural region with department fixed effects derived from UBIGEO. Supplementary Table S8 presents a complementary interaction analysis using continuous survey year, household poverty status, and their interaction.

Multicollinearity among covariates included in the adjusted model was assessed using variance inflation factors calculated from the dummy-coded predictor matrix. VIF values below 4 were considered acceptable and interpreted as indicating no problematic multicollinearity. All statistical tests were two-sided, and statistical significance was set at p < 0.05. Prevalence ratios and confidence intervals were rounded to two decimal places, except for estimates close to the null value, which were reported with three decimals when necessary to avoid ambiguity.

### Ethical considerations

This study used publicly available, de-identified secondary data from the Peruvian National Household Survey (ENAHO), conducted by the National Institute of Statistics and Informatics (INEI). The study protocol was reviewed and approved by the Institutional Committee for Ethics and Scientific Integrity (Comité Institucional de Ética e Integridad Científica, CIEIC) of Universidad Privada Norbert Wiener, Lima, Peru (Exp. No. A1301-2026; 16 July 2026). The study was conducted in accordance with the ethical principles of the Declaration of Helsinki and applicable Peruvian regulations. The analysis involved no direct contact with participants and no access to personally identifiable information. Informed consent for the original survey was obtained through INEI’s data collection procedures, and no additional consent was sought for this secondary analysis of anonymised public-use data.

## Results

The analytic sample comprised 330,165 adults aged 18 years or older who reported a recent health problem or need for care. Women represented 59.5% of the weighted population; 36.3% were aged 40–59 years, and 29.9% were aged 60 years or older. Primary education was the most common educational level (41.6%), 78.9% lived in urban areas, and 53.5% were insured through the Comprehensive Health Insurance scheme (SIS). Overall, 20.3% lived in households classified as poor, 24.1% reported Quechua, Aymara, or another indigenous language as their mother tongue, and 66.1% had a chronic illness or discomfort. IPRESS utilisation was reported by 41.2% of adults with a recent need for care (Table 1).

Among the 193,225 adults who did not use an IPRESS, 138,104 (weighted 67.4%) reported not seeking care, while 55,121 (32.6%) reported at least one non-IPRESS care-seeking pathway. Pharmacy or drugstore contact alone was the most frequently reported alternative (30.3%), followed by another place of care (1.3%) and care at home (0.7%). A small proportion reported multiple non-IPRESS care options (0.2%) (Supplementary Table S9).

Between 2015 and 2024, overall weighted IPRESS utilisation showed an annual relative change of −2.00% (95% CI, −2.25 to −1.75). Significant downward trends were also observed among non-poor adults (−1.69% per year; 95% CI, −1.95 to −1.43), adults in non-extreme poverty (−2.10%; 95% CI, −2.75 to −1.43), and adults in extreme poverty (−2.13%; 95% CI, −3.40 to −0.85). Annual prevalence declined sharply in 2020 and recovered only partially thereafter, while remaining consistently highest among non-poor adults and lowest among adults in extreme poverty (Figure 1).

In bivariate analyses, weighted IPRESS utilisation was 43.6% among non-poor adults, 32.3% among those in non-extreme poverty, and 28.5% among those in extreme poverty. Compared with non-poor adults, the crude prevalence of utilisation was 26% lower among adults in non-extreme poverty (PR, 0.74; 95% CI, 0.72–0.75) and 35% lower among those in extreme poverty (PR, 0.65; 95% CI, 0.63–0.68). Utilisation was also lower among adults with an indigenous mother tongue, rural residents, residents of the Highlands, and uninsured adults. Conversely, utilisation was higher among women, older adults, adults with higher education, insured adults, those with a chronic illness or discomfort, and residents of Metropolitan Lima (Table 2).

After multivariable adjustment, household poverty remained independently associated with lower IPRESS utilisation. In the fully adjusted model, adults in non-extreme poverty had a 20% lower prevalence of utilisation (aPR, 0.80; 95% CI, 0.79–0.82), while those in extreme poverty had a 26% lower prevalence (aPR, 0.74; 95% CI, 0.71–0.77), compared with non-poor adults. All types of health insurance were associated with greater utilisation than being uninsured, with the largest associations observed for private or other insurance (aPR, 2.08; 95% CI, 1.97–2.19), Armed Forces or Police insurance (aPR, 2.03; 95% CI, 1.94–2.14), and EsSalud (aPR, 2.01; 95% CI, 1.96–2.06). SIS coverage was associated with an 81% higher prevalence of utilisation (aPR, 1.81; 95% CI, 1.77–1.85).

Higher adjusted utilisation was also observed among women (aPR, 1.11; 95% CI, 1.10–1.13), adults aged 60 years or older (aPR, 1.13; 95% CI, 1.10–1.16), those with higher education (aPR, 1.16; 95% CI, 1.13–1.19), those with a chronic illness or discomfort (aPR, 1.16; 95% CI, 1.14–1.18), rural residents (aPR, 1.06; 95% CI, 1.04–1.07), and residents of Metropolitan Lima (aPR, 1.14; 95% CI, 1.12–1.16). In contrast, adults with an indigenous mother tongue (aPR, 0.97; 95% CI, 0.95–0.99) and residents of the Highlands (aPR, 0.89; 95% CI, 0.87–0.90) had lower utilisation. Disability, residence in the Rainforest, and age between 30 and 59 years were not independently associated with IPRESS utilisation in the fully adjusted model (Table 3).

The association between household poverty and IPRESS utilisation differed significantly across survey periods (global poverty-by-period interaction, P < 0.001). Compared with non-poor adults, utilisation was lower among adults in non-extreme and extreme poverty during 2015–2019 (aPR, 0.83; 95% CI, 0.81–0.86 and aPR, 0.80; 95% CI, 0.76–0.84, respectively), 2020–2021 (aPR, 0.74; 95% CI, 0.70–0.78 and aPR, 0.61; 95% CI, 0.54–0.70), and 2022–2024 (aPR, 0.78; 95% CI, 0.75–0.81 and aPR, 0.73; 95% CI, 0.68–0.79) (Table 4). Adjusted predicted prevalences showed a decline across all poverty groups during 2020–2021, followed by partial recovery in 2022–2024. Nevertheless, utilisation remained lower among poor adults in every period, with the widest separation observed during 2020–2021 (Figure 2).

## Discussion

This nationally representative repeated cross-sectional study found that fewer than half of Peruvian adults with a recent need for care used an IPRESS, and utilisation declined over the 2015–2024 period, with the sharpest reduction during 2020–2021 and only partial recovery thereafter. Poverty-related inequalities persisted throughout the study period and were most pronounced in 2020–2021, suggesting that economically disadvantaged households were particularly vulnerable to disruptions in access to formal care. Although health insurance was the factor most strongly associated with utilisation, the poverty gradient remained after insurance type was considered, indicating that financial coverage alone does not remove the indirect, organisational, geographic, and social barriers that shape effective access. The lower utilisation among adults with an indigenous mother tongue and residents of the Highlands further suggests that socioeconomic disadvantage overlaps with cultural and territorial inequities. Conversely, the higher utilisation observed among older adults and those with chronic conditions is likely to reflect greater healthcare need rather than more equitable access. Taken together, these findings indicate that the expansion of health insurance coverage in Peru has not yet translated into equitable use of formal health services among adults who report needing care.

The finding that fewer than half of adults with a recent need for care used formal health services indicates a substantial gap between perceived healthcare need and effective service utilisation in Peru. Overall, IPRESS use was 41.2%, with an estimated annual relative decline of 2.00% between 2015 and 2024, a marked reduction during 2020–2021, and only partial recovery thereafter. This prevalence exceeds the 25.4% frequency of physician consultation reported among Peruvians with a recent health problem in 2019, plausibly because IPRESS use encompasses more formal providers than physician-attended consultations [10].

The decline in formal healthcare use shown in Figure 1 may reflect both longstanding limitations in service delivery and changes in care-seeking behaviour, particularly during the COVID-19 pandemic. Before the pandemic, fragmentation, chronic underinvestment, and shortages of health professionals and equipment had already constrained Peru’s ability to provide timely care [8, 9]. During 2020–2021, the prioritisation of COVID-19 services and interruptions to routine consultations may have further reduced service availability, while transport restrictions, fear of infection, and financial hardship may have discouraged attendance [16, 17, 20]. An analysis of Peruvian administrative records documented 1,782,446 fewer chronic-disease service users than expected during the first pandemic month, followed by a gradual rather than immediate recovery [20]. Persistent resource constraints, postponed consultations, and uneven service capacity across regions may help explain the incomplete recovery after 2021. However, ENAHO did not provide comparable annual measures of facility staffing, consultation capacity, medicine availability, or service interruptions. We therefore cannot determine the relative contribution of healthcare supply constraints and changes in individual care-seeking behaviour to the observed decline.

The persistent poverty gradient observed in this study is consistent with evidence that economically disadvantaged populations experience lower effective access to healthcare even within systems that have expanded nominal insurance coverage. Compared with non-poor adults, IPRESS use remained lower among non-extreme poor adults (aPR 0.80) and extreme poor adults (aPR 0.74) after adjustment for insurance and other covariates. Annual declines were also steeper in these groups (−2.10% and −2.13%) than among non-poor adults (−1.69%). The gradient widened during 2020–2021: the corresponding aPRs changed from 0.83 and 0.80 in 2015–2019 to 0.74 and 0.61 during the pandemic, before recovering only partly to 0.78 and 0.73 in 2022–2024. This extends the Peruvian finding of a pro-rich concentration in physician consultation (Erreygers concentration index, 0.199) [10] and multicountry evidence that Peru retained a 20% relative income gap in barriers to seeking care [3]. Across Latin America and the Caribbean, households in the lowest wealth group were 27.3% more likely to report disrupted care than those in the highest group in 2021 [21]. Transport costs, forgone earnings, waiting time, medicine shortages, and limited capacity to substitute private care probably became more consequential during disruption. The significant poverty-by-period interaction therefore identifies unequal vulnerability to health-system shocks and supports equity-focused recovery and preparedness policies.

Although health insurance coverage exceeded 80% in our analytic sample, only 41.2% of adults reporting a recent health problem used an IPRESS during the preceding four weeks. These figures reflect different dimensions of access: insurance enrolment and actual service use. They should not be interpreted as evidence that every person who did not seek care had an unmet healthcare need. Nevertheless, the persistent poverty gradient suggests that insurance coverage alone does not remove the difficulties people face when seeking care. Even when consultations are covered, patients may still incur transportation costs, lose income while attending appointments, or pay for medicines and diagnostic tests that are unavailable at their designated facilities. These expenses can be particularly difficult for households living in poverty. Limited appointment availability, long waiting times, workforce shortages, medicine stockouts, and referral difficulties may also discourage or delay formal care. Such constraints may vary across Peru’s fragmented healthcare networks. In our adjusted analysis, IPRESS use was higher among adults covered by EsSalud (aPR, 2.01), SIS (aPR, 1.81), Armed Forces/Police insurance (aPR, 2.03), and private or other insurance (aPR, 2.08) than among uninsured adults. Previous Peruvian studies support the positive association between insurance and service use [11, 14], while regional evidence highlights persistent gaps in healthcare quality [7]. Because these specific barriers were not directly examined in our analysis, they should be considered plausible explanations rather than established causes of non-use.

The absence of formal healthcare use does not necessarily mean that individuals received no care. Our additional analysis showed that 67.4% of adults who did not use an IPRESS reported not seeking care, whereas 32.6% reported at least one alternative care-seeking pathway. Pharmacy or drugstore contact was the most common alternative, while care at home and other places accounted for much smaller proportions. These findings suggest that formal service non-use reflects different care-seeking pathways rather than a single pattern of foregoing care. Pharmacy-based contact should not be equated with unregulated or traditional care, and home-based care does not necessarily indicate self-medication. Similarly, not seeking care does not establish that a clinically assessed need remained unmet. Our analytic sample excluded respondents who considered medical attention unnecessary because their condition was not serious, as well as those reporting both formal facility and pharmacy use. Although cultural preferences, language barriers, and previous experiences of mistreatment may influence care-seeking decisions, their role in the use of alternative care was not directly examined. These distinctions are important when interpreting socioeconomic inequalities in healthcare utilisation.

The lower utilisation observed among adults living in the Highlands and those with a Quechua, Aymara, or other Indigenous mother tongue suggests that territorial and language-related inequalities persist after adjustment for household poverty, health insurance, and other covariates. IPRESS use was lower among Highlands residents (aPR, 0.89) and Indigenous-language speakers (aPR, 0.97), but higher in Metropolitan Lima (aPR, 1.14). Similar differences were reported in ENAHO 2019, when 21.6% of native-language speakers received a physician consultation compared with 26.6% of non-native-language speakers [10]. Another Peruvian study associated Quechua ethnicity, Aymara ethnicity, and speaking a native language with non-use attributed to mistreatment (aPRs, 2.60, 2.98, and 2.28, respectively) [6]. Median rural travel times to primary, secondary, and tertiary healthcare facilities were estimated at 1.9, 2.3, and 2.2 times those in urban areas, respectively [22]. In our analysis, rural residence was associated with lower utilisation before adjustment (PR, 0.86) but slightly higher utilisation after adjustment (aPR, 1.06). This reversal should not be interpreted as evidence of better rural access, as it may reflect differences in the distribution of poverty, insurance, Indigenous-language speakers, and regional disadvantage.

Several barriers may help explain these findings. In geographically isolated communities, long journeys, limited road connectivity, and costly transportation can make health facilities difficult to reach, even when consultations are covered by insurance [12, 22]. Travelling for care may also mean losing income or time needed for household responsibilities. For people whose first language is Quechua, Aymara, or another Indigenous language, communication difficulties, limited access to interpretation services, and previous experiences of disrespectful treatment may discourage formal consultations [6]. Such barriers could influence decisions to visit pharmacies, seek home-based assistance, or rely on traditional care. However, our study did not assess whether cultural or territorial characteristics were associated with these alternative care-seeking pathways. Improving local service availability, transport and referral arrangements, and culturally respectful communication may help reduce these inequalities.

Higher utilisation among older adults and individuals with chronic conditions is likely to reflect greater and more frequent healthcare needs rather than preferential access to services. Adults aged 60 years or older and those reporting chronic illness or discomfort had higher adjusted IPRESS use than their reference groups (aPR 1.13 and 1.16), while women also used services more frequently than men (aPR 1.11). Similar patterns were reported in Peru in 2019, when physician consultation reached 27.9% among women versus 22.2% among men and chronic disease contributed to socioeconomic inequality in use [10]. Among Peruvians with COVID-19 symptoms, adults aged 50–59 and ≥60 years had lower non-use than younger adults (aPR 0.72 and 0.74), suggesting that perceived severity and clinical risk can intensify care seeking [14]. Quasi-experimental evidence also showed that the utilisation effect of SIS was particularly large among older adults, at 13 percentage points [11]. However, the pandemic-related collapse in chronic-disease services demonstrates that higher underlying need does not protect these groups from supply disruption [20]. Women’s higher use may reflect differences in morbidity recognition, preventive and reproductive needs, and help-seeking behaviour. Higher contact rates should therefore not be interpreted as better health or adequate access; assessment should determine whether use is proportionate to need and produces timely, continuous, and effective care.

The positive educational gradient and modest associations for marital status suggest that informational and social resources may facilitate navigation of the Peruvian health system. Compared with adults without formal education, adjusted IPRESS use increased among those with primary, secondary, and higher education (aPRs 1.06, 1.09, and 1.16). This agrees with evidence from Peruvians with COVID-19 symptoms, among whom secondary and higher education were associated with lower non-use (aPR 0.67 for each level) [14], and with national evidence that physician consultation was concentrated among better-educated groups [10]. Conversely, the larger SIS effect among adults with lower education 6.4 percentage points suggests that insurance can partly offset educational barriers [11]. Relative to single adults, use was modestly higher among cohabiting, married, widowed, and divorced or separated adults (aPRs 1.03, 1.05, 1.04, and 1.04), possibly reflecting social support, although this pathway was not measured. Adults aged 30–59 years, Rainforest residents, and people with disability showed no independent association. The disability result should not be equated with equity: a regional systematic review found that people with disabilities generally used more services, plausibly reflecting greater need, while evidence on affordability and quality remained limited [23]. The Rainforest null estimate may also conceal substantial within-region heterogeneity. These findings distinguish equal contact prevalence from equitable, need-responsive access.

This study makes three related scientific contributions. First, it provides a decade-long, nationally representative assessment of IPRESS use specifically among adults with a recent healthcare need, thereby distinguishing effective service contact from insurance enrolment or utilisation measured in the general population. Second, it shows that socioeconomic inequality was dynamic rather than fixed: the poverty gradient widened during 2020–2021 and narrowed only partially thereafter, identifying differential resilience to health-system disruption as an additional dimension of inequality. Third, the simultaneous consideration of household poverty status, type of health insurance, mother tongue/language, natural region, and need-related characteristics clarifies how financial, institutional, territorial, and cultural barriers overlap within Peru’s segmented health system. Within the ENAHO sampling framework, the estimates are generalisable to adults aged ≥18 years living in private households in Peru who reported a recent health problem and had classifiable IPRESS use. The findings may also be analytically informative for middle-income countries with fragmented insurance arrangements, marked territorial inequalities, and pandemic-related service disruption. However, transferability beyond Peru is conceptual rather than numerical, and the estimates should not be extrapolated to adults without perceived healthcare need, institutionalised populations, children, or health systems with substantially different service-delivery structures.

The principal strengths of this study are its large nationally representative sample of 330,165 adults, the harmonisation of 10 consecutive ENAHO rounds, and the restriction of the denominator to individuals reporting a recent healthcare need. All estimates incorporated sampling weights, strata, and clusters, while modified Poisson regression generated directly interpretable prevalence ratios for a common outcome. Furthermore, the poverty-by-period interaction enabled assessment of whether socioeconomic inequalities changed during pandemic disruption and subsequent recovery rather than assuming that the gradient remained constant.

Several limitations should nevertheless be considered. The repeated cross-sectional design describes population-level changes and cannot establish within-person trajectories or causal relationships. Recent health problems and IPRESS use were self-reported and may be affected by recall error, differential recognition of illness, or outcome misclassification. Perceived need was not standardised according to clinical severity, and an IPRESS contact does not establish timely attendance, continuity, technical quality, diagnostic resolution, or health benefit. The exclusion of respondents who considered care unnecessary or their condition not serious, as well as those reporting both formal facility and pharmacy use, also warrants consideration. These restrictions define a selected analytic population and may affect estimates of formal healthcare utilisation if care-seeking patterns differ across socioeconomic groups. Consequently, our findings should not be generalised to all adults reporting a recent health problem without considering these eligibility criteria.

The additional descriptive analysis distinguished pharmacy or drugstore contact, care at home, other non-IPRESS care, and no care sought. However, it could not determine whether pharmacy-based or home-based care involved self-medication, or whether individuals who did not seek care had an unmet clinical need. Detailed reasons for not seeking care were not otherwise examined. The available variables did not fully capture travel and waiting times, indirect costs, medicine availability, provider supply, cultural safety, or personal care preferences; therefore, residual confounding remains possible. Survey administration and reporting behaviour may also have changed during the pandemic. Finally, although the adjusted reversal of the rural association is compatible with statistical suppression by correlated socioeconomic and territorial factors, the available data cannot identify a single mechanism explaining this pattern.

## Conclusion

Among Peruvian adults reporting a recent need for care, fewer than half used IPRESS during 2015–2024, and utilisation declined over time. The marked reduction during 2020–2021 and incomplete recovery thereafter indicate a sustained health-system disruption rather than a brief interruption. Household poverty remained independently associated with lower IPRESS use, while the widening gradient during the pandemic demonstrates that health-system shocks disproportionately restricted effective access among non-extreme poor and extreme poor adults. Although every insurance scheme was associated with greater use than being uninsured, persistent inequalities by poverty status, Indigenous mother tongue, and residence in the Highlands show that insurance affiliation alone does not ensure timely and effective care. Higher utilisation among women, older adults, and individuals with chronic illness or discomfort most likely reflects greater healthcare need and should not automatically be interpreted as preferential access. Achieving equitable universal health coverage in Peru therefore requires monitoring actual service use among people in need, strengthening resilient primary care, reducing territorial and cultural barriers, and ensuring that insurance entitlements are matched by adequate providers, medicines, referral capacity, and continuity of care.

## Figures and Tables

**Table:** 

aPR	Adjusted prevalence ratio
CI	Confidence interval
COVID-19	Coronavirus disease 2019
ENAHO	Peruvian National Household Survey on Living Conditions and Poverty (*Encuesta Nacional de Hogares sobre Condiciones de Vida y Pobreza*)
EsSalud	Social Health Insurance
INEI	National Institute of Statistics and Informatics of Peru (*Instituto Nacional de Estadística e Informática*)
IPRESS	Health Service Provider Institution (*Institución Prestadora de Servicios de Salud*)
PR	Prevalence ratio
PSU	Primary sampling unit
SIS	Comprehensive Health Insurance (*Seguro Integral de Salud*)
UBIGEO	Geographic location code
UHC	Universal health coverage
UI	Uncertainty interval
VIF	Variance inflation factor

## Data Availability

The ENAHO microdata, questionnaires, methodological documents, and technical files are publicly available through the INEI microdata portal. This study used de-identified public-use data from the 10 annual ENAHO rounds conducted between 2015 and 2024. The analytical dataset was constructed by harmonising annual survey files using consistent individual and household identifiers, sampling weights, strata, primary sampling units, recent morbidity indicators, formal health service utilisation variables, household poverty classifications, health insurance categories, and sociodemographic, health-related, and territorial covariates. The harmonised analytical dataset and statistical code generated for this study may be obtained from the corresponding author upon reasonable request and subject to appropriate academic and data-management conditions. The original ENAHO datasets remain subject to INEI’s terms of use.

## Patient and Public Involvement

Patients and members of the public were not directly involved in the design, conduct, analysis, interpretation, reporting, or dissemination planning of this study. The research was based exclusively on the secondary analysis of publicly available, de-identified ENAHO microdata and involved no direct interaction with participants, relatives, caregivers, or community representatives.

## Artificial Intelligence Assistance Statement

Artificial intelligence-assisted tools were used to support language editing, formatting alignment, and manuscript organisation. These tools were not used for data collection, dataset construction, variable definition, statistical analysis, generation of statistical estimates, or independent interpretation of the findings.

All results presented in the tables and figures were generated from the harmonised ENAHO 2015–2024 dataset using prespecified variable definitions, individual sampling weights, strata, primary sampling units, and survey-adjusted statistical models. The authors reviewed all AI-assisted edits, verified the accuracy and consistency of the manuscript against the underlying data and statistical outputs, approved the final version, and take full responsibility for its integrity and content.

## Additional File

The additional file for this article can be found as follows:
