# Supplementary material for: Trends in Socioeconomic Inequalities in the Use of Formal Health Services Among Peruvian Adults in Need of Care: 2015–2024
Source: Ann Glob Health. 2026 Nov 10;92(1):110. doi: 10.5334/aogh.5481 (PMC13761779; doi:10.5334/aogh.5481)
Supplement: Supplementary file 1 [file agh-92-1-5481-s1.pdf]

Table S1. Variables used, operational definitions, and recoding rules

Variable in manuscript	Operational definition	Original ENAHO variable/recode name	Final categories/coding	Reference category
**Recent need for care / recent health problem**	Criterion used to define the analytic population. Adults were included if they reported any health problem, illness, discomfort, relapse, accident, or other health-related condition in the previous four weeks.	Original ENAHO morbidity items p4021–p4024; harmonized before constructing the analytic file	Included = any recent health problem reported; excluded = no recent health problem. The final analytic population included adults aged ≥18 years with recent need for care.	Not applicable
**IPRESS use**	Main outcome. Use of formal health services/IPRESS among adults reporting recent need for care.	Harmonized variable nusfps, created from original care-seeking items p4031–p40313 and provider items p4041–p4047	0 = Used formal health services/IPRESS; 1 = Did not use formal health services/IPRESS	Did not use IPRESS
**Household poverty status**	Main socioeconomic exposure based on INEI household poverty classification.	pobreza	Non-poor; Non-extreme poor; Extreme poor	Non-poor
**Survey year**	Calendar year of ENAHO survey.	anio	2015–2024	2015
**Survey period**	Analytical period created from survey year.	anio	2015–2019; 2020–2021; 2022–2024	2015–2019
**Sex**	Sex recorded in ENAHO.	p207	Male; Female	Male
**Age group**	Age in completed years categorized into epidemiological groups.	p208a	18–29; 30–39; 40–59; ≥60 years	18–29 years
**Educational level**	Highest educational level harmonized across survey years.	nivel_educ	No formal education; Primary; Secondary; Higher	No formal education
**Marital status**	Civil/marital status recoded into five categories.	p209	Single; Cohabiting; Married; Widowed; Divorced/Separated	Single
**Mother tongue/language**	Language variable harmonized into Spanish versus indigenous language.	lengua	Spanish; Quechua/Aymara/Other indigenous	Spanish

**Type of health insurance**	Main health insurance scheme.	seguro_salud	Uninsured; EsSalud; SIS; Armed Forces/Police; Private/Other	Uninsured
**Chronic illness or discomfort**	Self-reported chronic illness or chronic discomfort.	enf_cronica	No; Yes	No
**Disability**	Any reported disability dimension.	discapacidad	No; Yes	No
**Area of residence**	Urban/rural residence.	area	Urban; Rural	Urban
**Natural region**	Natural geographic region.	region_nat	Coast; Highlands; Rainforest; Metropolitan Lima	Coast
**Department**	Department derived from the first two digits of UBIGEO. Used in sensitivity analysis.	ubigeo	Department/administrative region fixed effects derived from UBIGEO	Lima in sensitivity model
**Sampling weight**	Individual expansion factor for population estimates.	facpob07	Continuous survey weight	Not applicable
**Stratum**	Survey stratum.	estrato	Used for complex survey design	Not applicable
**Cluster/PSU**	Primary sampling unit.	conglome	Used for complex survey design	Not applicable

Note: The analytic population comprised adults aged 18 years or older who reported symptoms, illness, relapse of a chronic condition, or an accident during the preceding four weeks (p4021–p4024). Respondents who reported that their condition was not serious or care was unnecessary (p4095) were excluded. Respondents reporting both formal healthcare facility use (any of p4031–p4039) and pharmacy care (p40310) were also excluded to retain exclusive care-seeking patterns. The final dataset contained 330,165 adult observations across the 2015–2024 ENAHO survey rounds.

Table S2. Variance inflation factors for covariates included in the adjusted model

Covariate term	VIF
**Household poverty status: Non-extreme poor (ref Non-poor)**	1.12
**Household poverty status: Extreme poor (ref Non-poor)**	1.08
**Sex: Female (ref Male)**	1.10
**Age group: 30–39 years (ref 18–29 years)**	1.94
**Age group: 40–59 years (ref 18–29 years)**	3.04
**Age group: ≥60 years (ref 18–29 years)**	3.78
**Educational level: Primary (ref No formal education)**	3.59
**Educational level: Secondary (ref No formal education)**	3.87
**Educational level: Higher (ref No formal education)**	3.87
**Marital status: Cohabiting (ref Single)**	2.45

**Marital status: Married (ref Single)**	3.11
**Marital status: Widowed (ref Single)**	2.10
**Marital status: Divorced/Separated (ref Single)**	1.90
**Mother tongue/language: Quechua/Aymara/Other indigenous (ref Spanish)**	1.38
**Type of health insurance: EsSalud (ref Uninsured)**	2.06
**Type of health insurance: SIS (ref Uninsured)**	2.02
**Type of health insurance: Armed Forces/Police (ref Uninsured)**	1.07
**Type of health insurance: Private/Other (ref Uninsured)**	1.07
**Chronic illness or discomfort: Yes (ref No)**	1.20
**Disability: Yes (ref No)**	1.12
**Area of residence: Rural (ref Urban)**	1.40
**Natural region: Highlands (ref Coast)**	1.85
**Natural region: Rainforest (ref Coast)**	1.46
**Natural region: Metropolitan Lima (ref Coast)**	1.26
**Survey year dummy terms: 2016–2024 (ref 2015), range**	1.61–1.88

Note: All VIF values were below 4, indicating no evidence of problematic multicollinearity among covariates included in the adjusted model.

Table S3. Adjusted association between survey period and IPRESS use among Peruvian adults reporting a recent health problem

Period	Adjusted PR	95% CI	p-value
**2015–2019**	1.00	Reference	—
**2020–2021**	0.73	0.72–0.75	<0.001
**2022–2024**	0.86	0.85–0.87	<0.001

Note: Modified Poisson regression adjusted for household poverty status, sex, age group, educational level, marital status, mother tongue/language, type of health insurance, chronic illness or discomfort, disability, area of residence, and natural region. PR: prevalence ratio; CI: confidence interval.

Table S4. Year-specific weighted prevalence of IPRESS use overall and by household poverty status

Year	Overall IPRESS use (%)	Non-poor (%)	Non-extreme poor (%)	Extreme poor (%)
**2015**	46.3	48.4	37.8	31.9
**2016**	43.8	45.9	34.2	34.3
**2017**	43.2	45.1	35.7	30.8
**2018**	44.4	46.5	34.5	31.6
**2019**	45.3	47.1	36.3	32.6
**2020**	31.2	34.0	22.4	18.8
**2021**	34.1	36.5	26.4	19.7
**2022**	38.2	41.1	30.2	24.8
**2023**	41.3	44.3	32.9	30.3
**2024**	40.1	42.7	32.2	28.8

Note: Percentages are survey-weighted using facpob07. The denominator is restricted to adults aged ≥18 years with recent need for care.

Table S5. Weighted prevalence of IPRESS use by period and household poverty status

Period	Overall IPRESS use (%)	Non-poor (%)	Non-extreme poor (%)	Extreme poor (%)	Absolute gap: non-poor vs extreme poor (pp)	Relative ratio: non-poor/extreme poor
**2015–2019**	44.6	46.6	35.7	32.3	14.3	1.44
**2020–2021**	32.7	35.2	24.3	19.2	16.0	1.83
**2022–2024**	39.9	42.7	31.8	28.2	14.6	1.52

Note: pp: percentage points. The periods represent pre-pandemic years, pandemic disruption, and post-2022 partial recovery.

Table S6. Annual absolute and relative socioeconomic gaps in IPRESS use

Year	Non-poor IPRESS use (%)	Non-extreme poor (%)	Extreme poor (%)	Absolute gap non-poor vs extreme poor (pp)	Relative ratio non-poor/extreme poor	Absolute gap non-poor vs non-extreme poor (pp)	Relative ratio non-poor/non-extreme poor
**2015**	48.4	37.8	31.9	16.5	1.52	10.6	1.28
**2016**	45.9	34.2	34.3	11.5	1.34	11.6	1.34
**2017**	45.1	35.7	30.8	14.3	1.46	9.4	1.26
**2018**	46.5	34.5	31.6	14.9	1.47	11.9	1.34
**2019**	47.1	36.3	32.6	14.6	1.45	10.8	1.30
**2020**	34.0	22.4	18.8	15.2	1.81	11.6	1.52
**2021**	36.5	26.4	19.7	16.7	1.85	10.0	1.38
**2022**	41.1	30.2	24.8	16.2	1.65	10.8	1.36
**2023**	44.3	32.9	30.3	13.9	1.46	11.4	1.35
**2024**	42.7	32.2	28.8	13.9	1.48	10.5	1.33

Note: pp: percentage points. Ratios greater than 1 indicate higher IPRESS use among non-poor adults compared with poorer groups. Percentages are survey-weighted using facpob07. The denominator is restricted to adults aged ≥18 years with recent need for care.

Table S7. Sensitivity analysis using department as an alternative territorial adjustment

Model specification	Household poverty status	aPR (95% CI)	p-value
**Main model: area + natural region**	Non-poor	Reference	—
	Non-extreme poor	0.80 (0.79–0.82)	<0.001
	Extreme poor	0.74 (0.71–0.77)	<0.001
**Sensitivity model: area + department fixed effects**	Non-poor	Reference	—
	Non-extreme poor	0.81 (0.79–0.82)	<0.001
	Extreme poor	0.73 (0.70–0.76)	<0.001

Note: The main model used area and natural region to preserve comparability and avoid overparameterization. The sensitivity model replaced natural region with department fixed effects derived from ubigeo, while retaining area of residence and the remaining covariates.

Table S8. Supplementary interaction analysis using continuous survey year

Household poverty status	Poverty aPR at 2015 (95% CI)	p-value	Annual relative change PR (95% CI)	p-value
**Non-poor**	Reference	—	0.975 (0.973–0.978)	<0.001
**Non-extreme poor**	0.83 (0.81–0.86)	<0.001	0.966 (0.960–0.972)	<0.001
**Extreme poor**	0.78 (0.73–0.83)	<0.001	0.967 (0.954–0.979)	<0.001
**Interaction term**			**Interaction PR (95% CI)**	**p-value**
**Non-extreme poor × continuous year**			0.990 (0.984–0.997)	0.005
**Extreme poor × continuous year**			0.991 (0.979–1.004)	0.178
**Overall poverty × continuous year interaction**			—	0.011

Note: The model included continuous survey year centered at 2015, household poverty status, and their interaction. It adjusted for sex, age group, educational level, marital status, mother tongue/language, type of health insurance, chronic illness or discomfort, disability, area of residence, and natural region. PR values below 1 indicate declining IPRESS use over time.

Table S9. Care-seeking pathways among adults who did not use formal health services, ENAHO 2015–2024

Care-seeking pathway	n	Weighted %
**Did not seek care**	138,104	67.42
**Pharmacy or drugstore only**	51,223	30.32
**Care at home only**	1,076	0.73
**Another place of care only**	2,561	1.33
**Multiple non-IPRESS care options**	261	0.20
**Total**	193,225	100.00

Note: Categories are mutually exclusive. Multiple non-IPRESS care options refer to respondents reporting more than one of the following: pharmacy or drugstore, care at home, or another place of care. Unweighted counts and survey-weighted percentages are presented. The denominator includes 193,225 adults who did not use an IPRESS after applying the study eligibility criteria. Percentages were calculated using the annual ENAHO sampling weights.
